# Reproducible image-based profiling with Pycytominer

**Published:** 2023-11-22

**Authors:** Erik Serrano, Srinivas Niranj Chandrasekaran, Dave Bunten, Kenneth I. Brewer, Jenna Tomkinson, Roshan Kern, Michael Bornholdt, Stephen Fleming, Ruifan Pei, John Arevalo, Hillary Tsang, Vincent Rubinetti, Callum Tromans-Coia, Tim Becker, Erin Weisbart, Charlotte Bunne, Alexandr A. Kalinin, Rebecca Senft, Stephen J. Taylor, Nasim Jamali, Adeniyi Adeboye, Hamdah Shafqat Abbasi, Allen Goodman, Juan C. Caicedo, Anne E. Carpenter, Beth A. Cimini, Shantanu Singh, Gregory P. Way

**Affiliations:** 1. Department of Biomedical Informatics, University of Colorado School of Medicine; 2. Imaging Platform, Broad Institute of MIT and Harvard; 3. Independent Researcher; 4. Case Western Reserve University; 5. Data Sciences Platform, Broad Institute of MIT and Harvard; 6. Genentech gRED; 7. Morgridge Institute for Research, University of Wisconsin-Madison

## Abstract

Technological advances in high-throughput microscopy have facilitated the acquisition of cell images at a rapid pace, and data pipelines can now extract and process thousands of image-based features from microscopy images. These features represent valuable single-cell phenotypes that contain information about cell state and biological processes. The use of these features for biological discovery is known as image-based or morphological profiling. However, these raw features need processing before use and image-based profiling lacks scalable and reproducible open-source software. Inconsistent processing across studies makes it difficult to compare datasets and processing steps, further delaying the development of optimal pipelines, methods, and analyses. To address these issues, we present Pycytominer, an open-source software package with a vibrant community that establishes an image-based profiling standard. Pycytominer has a simple, user-friendly Application Programming Interface (API) that implements image-based profiling functions for processing high-dimensional morphological features extracted from microscopy images of cells. Establishing Pycytominer as a standard image-based profiling toolkit ensures consistent data processing pipelines with data provenance, therefore minimizing potential inconsistencies and enabling researchers to confidently derive accurate conclusions and discover novel insights from their data, thus driving progress in our field.

## Introduction

In the past thirty years, high-content microscopy has undergone a remarkable technological transformation that has given scientists the ability to acquire thousands of single-cell measurements in high-throughput experiments.^1–4^ This deluge of microscopy data greatly expands opportunities to study cell biology, particularly from a systems biology perspective. In turn, there has been a proliferation of open-source software tools tailored to image analysis, including Fiji^5^, CellProfiler^6^, QuPath^7^, napari^8^, and others. While these tools can rapidly derive biological insights from large microscopy datasets, they lack downstream bioinformatics processing of image-based features, which often must be transformed prior to data analysis. Recently, the field of image-based profiling has emerged to fill these gaps by performing bioinformatics data processing of cell features extracted from microscopy images.^3,9–11^ By capturing images of cells treated with perturbations such as chemicals or genetic agents, and extracting image-based features, researchers can analyze observed changes occurring in organelles, compare cell states, and develop new hypotheses for biological mechanisms.^11^

Thus far, the primary application for image-based profiling has been in drug development.^9,10,12^ Specifically, image-based profiling plays a central role in Phenotypic Drug Discovery (PDD), offering a route for disease phenotype discovery, target identification, drug repurposing, toxicity assessment, and the exploration of novel therapeutic hypotheses.^10,13^ For example, image-based profiling can determine if certain small molecules can drive disease cell states towards to healthy states.^14,15^ Furthermore, integrating image-based profiling with omics analyses enhances biological insights, as omics and cell imaging provide complementary information about cell states.^16–18^

Image-based profiling has provided novel biological insights, which have been uncovered through extensive data processing steps. However, there is an increasing demand for an actively maintained, open-source software toolkit that supports reproducible and scalable image-based profiling functionalities. In 2017, a consortium of experts established best practices, including feature extraction, data processing, and analysis.^3^ A subset of us initiated a library in R (called cytominer^19^), but later pivoted to Python (Pycytominer), finding its extensive ecosystem of machine learning and image processing libraries more conducive for development.^20^ The shift toward Python aligns with the current trend of the scientific and image analysis communities, where Python has gained widespread adoption due to its available packages for scientific software.^21^ Furthermore, other image-based profiling software tools like the open-source BioProfiling.jl^22^ address gaps in other programming languages while also implementing best practices. Similarly, StratoMineR is a commercially available, web-based tool built for analyzing high-dimensional morphological datasets from high-content screening experiments.^23^ Other software, like Squidpy^24^, also contains portions of image-based profiling for processing spatial data, placing a distinct focus on cell-type identification and gene expression. Earlier software packages have inspired current work and pursued similar goals, but maintenance has slowed over time and therefore these packages lack critical functionality and user support.^25^ Nevertheless, these projects have aided in establishing image-based profiling as a science that has already contributed to novel insights in cell biology and drug discovery.

Here we introduce Pycytominer, an open-source Python package for processing image-based profiles extracted from microscopy images of cells. This project resulted from community discussions and consensus stemming from our inaugural work together.^3^ Pycytominer is developed through the usage of Python^21^, Pandas^26^, Apache Parquet^27^, and SQLAlchemy^28^ to provide a reliable and user-friendly environment for working with image-based features. It uses a modular API, enabling flexible image-based profiling. Pycytominer is actively maintained by a community of users and developers. The project’s repository includes a clear code of conduct, usage documentation, and contributing guidelines, which outline the process for contributors who want to participate in package development. The repository also contains an extensive testing suite, along with continuous integration and continuous development (CI/CD) pipelines, to ensure the program’s stability. Currently, Pycytominer curates and processes image-based features extracted from CellProfiler and DeepProfiler^29^ (a deep learning complement to CellProfiler), and we have plans to expand support. Pycytominer is also actively used in several academic and industry labs; for example, scientists used Pycytominer to process two of the largest publicly-available Cell Painting^30^ datasets: Joint Undertaking in Morphological Profiling (JUMP)^31^ and Library of Integrated Network-Based Cellular Signatures (LINCS).^17^ Pycytominer is also used to process the majority of the currently 31 Cell Painting datasets in the Cell Painting Gallery.^32^ In this resource paper, we describe Pycytominer as the standard toolkit for image-based profiling. We describe the Pycytominer core API, available data processing functions, and how to orchestrate Pycytominer’s functionality into reproducible pipelines. Furthermore, we present Pycytominer community practices, establishing it as an adaptable and extensible tool for researchers studying image-based profiling.

## Results

### Generating the inputs for Pycytominer processing

**Figure 1.**
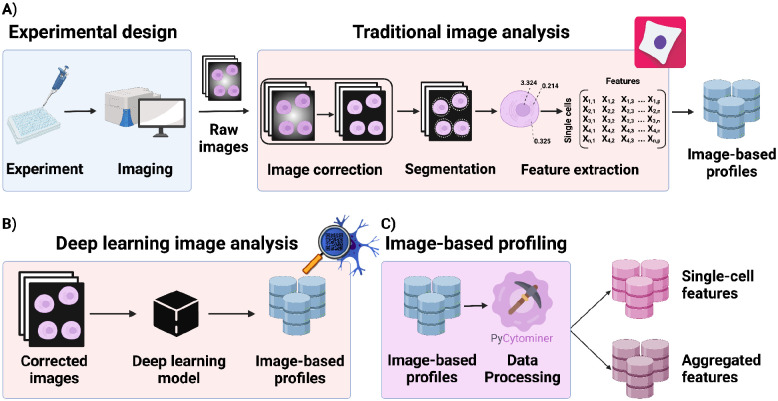
The standard image-based profiling experiment. **(A)** In the experimental design phase, a scientist plates cells, often perturbing them with chemical or genetic agents and performs microscopic imaging. In image analysis, using CellProfiler for example, a scientist applies several data processing steps to generate image-based profiles. In addition, scientists can also apply deep learning models using DeepProfiler, for example, to generate image-based profiles. **(C)** Pycytominer performs image-based profiling to process morphology features ready for downstream analyses.

Pycytominer processes image-based profiles, which are initially extracted from microscopy images. Image-based profiles are information-rich, providing unbiased measurements of cell morphology including cell shape, cell size, stain intensities, textures, and more, which give insights into biological mechanisms.^3,10^ Alternatively, profiles can be derived from deep learning-based networks used as feature extractors, trained by a rapidly advancing set of strategies such as self-supervised learning, vision transformers, and masked autoencoders.^33–37^ Either way, before scientists can use Pycytominer, they must first perform two core steps: (1) Data collection (including experimental design) and (2) image analysis (Figure 1A). Researchers first design an experiment, often subjecting cells to small molecule or genetic perturbations, and incubate them for a certain amount of time. Following an incubation period, cells can undergo staining, where fluorescence dyes are applied to mark specific cellular compartments.^10^ The most common image-based profiling assay is the Cell Painting assay.^30^ It is important to note that while fluorescence microscopy is a common approach, image-based profiling methods can extract morphologies from any microscopy image type. For example, brightfield imaging, which as a non-destructive technique, can be used for measuring dynamic processes, is becoming more common.^38^ We also expect that brightfield imaging will expand in conjunction with the development of virtual stain prediction techniques.^39^

After acquiring the microscopy images, scientists must perform image analysis. The raw microscopy images undergo a series of image processing algorithms to enhance image quality, perform cell segmentation, and extract morphology features.^3,10^ For example, images that are acquired through optical microscopy are known to have an uneven distribution of lighting on the images, such as vignetting,^3,40^ which can be corrected with (flat field) illumination correction.^41^ Next, single-cell segmentation algorithms produce binary masks, containing pixel information that distinguishes between cell objects and image background.^42^ The masks generated by segmentation algorithms, commonly generated by software like CellProfiler^43,44^ and CellPose^45^, provide boundaries of cells and subcellular structures within the images.^46^ Feature extraction algorithms use these masks to quantify diverse morphological characteristics per single cell, including cell shape, size, stain intensity, and texture.^9^ Alternatively, features can be extracted directly from the images without segmenting single-cells using, for instance, the application of a deep learning-based feature extractor directly on full corrected images (Figure 1B). Either way, image analysis results in a set of quantitative features that describe the observed cellular and subcellular properties.^9,46^ It is essential to process these features before carrying out custom analyses for various biological applications, and Pycytominer provides a standardized toolkit for processing these image-based profiles (Figure 1C).

### Pycytominer core interface and philosophy

**Figure 2.**
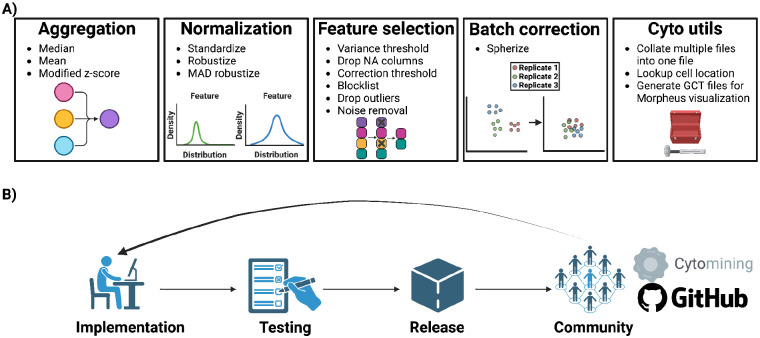
Pycytominer’s core Application Programming Interface (API) and software practices. (**A)** Pycytominer performs five fundamental functions, each implemented with a simple and intuitive API. Each function enables a user to implement various methods for executing operations. **(B)** The open-source Pycytominer community supports best development practices to facilitate code effectiveness and longevity.

Pycytominer provides user-friendly access to image-based profiling processing functions, allowing researchers to efficiently handle and derive insights from large-scale image-based datasets. It encompasses five core methods: Aggregation, Normalization, Feature Selection, Batch Correction, and Cyto Utilities, each of which plays a crucial role in the data processing pipelines (Figure 2A). Pycytominer applies these methods collectively to reduce dataset complexity, ensure consistent feature scaling, identify informative features, rectify batch effects, and offer custom functionality for CellProfiler^43,44^, DeepProfiler^33^, and Morpheus^47^ data. We elaborate on these core methods in Table 1.

We rigorously apply open-source best practices during Pycytominer’s development in four main categories: Implementation, testing, release, and community (Figure 2B). (1) *Implementation*. Pycytominer eases the process of contributing code by providing development container specifications usable in VSCode or GitHub Codespaces that contain the full set of software dependencies needed to develop and test the codebase. When changes are ready, contributors submit pull requests, which must be reviewed to ensure adherence to best practices such as modularization, code styling, and documentation. (2) *Testing*. Pycytominer’s comprehensive testing suite, including unit tests and code coverage analysis, serves as a crucial step to ensure the correctness and functionality of the software implementation. Testing every new change against the full test suite reduces the introduction of software bugs and ensures consistent behavior across versions. (3) *Release*. Pycytominer follows semantic versioning and maintains a changelog to ensure users are kept informed of new features and important changes. Releases are made available directly on GitHub and are also packaged for use within Python’s two major package repositories PyPI and conda. In addition, Pycytominer supports operating environment containerization (facilitated by Docker^48^), encapsulating dependencies to enhance reproducibility. (4) *Community*. Pycytominer cultivates our open-source community by welcoming new contributors with clear contributing instructions and guidelines and a code-of-conduct to ensure professional standards are kept. These community efforts are essential for good collaboration, maintaining quality, and ensuring project sustainability. Embracing the full set of these best software practices fosters a collaborative environment that facilitates continuous improvements, encourages reproducibility, welcomes newcomers, and contributes to package usability for developers and users alike.

### Pycytominer can facilitate analysis of publicly-available resources and outputs several intermediate data types

**Figure 3.**
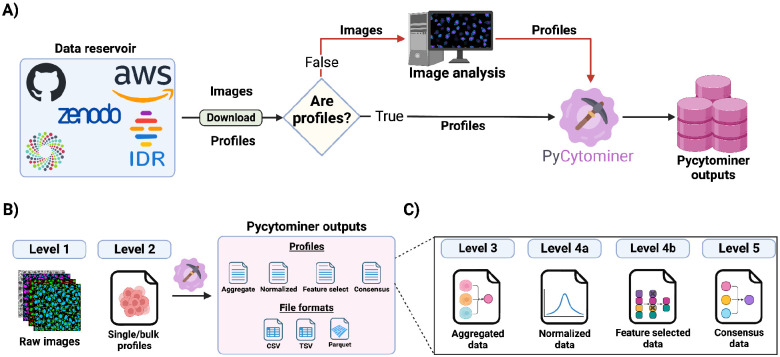
A data-centric view of Pycytominer workflows and outputs. **(A)** Pycytominer can be integrated into diverse workflows. The blue box illustrates a range of data repositories hosting both microscopy images and image-based profiles. Images must be analyzed to extract features prior to using Pycytominer for image-based profiling. Subsequently, downloaded image-based profiles can be directly sent to Pycytominer for image-based profiling. **(B)** Pycytominer generates a variety of profile formats like comma-separated values (CSV), tab-separated values (TSV), or parquet, which all support version control. **(C)** Pycytominer can output several intermediate data files (often termed “data levels”) which are aggregated, normalized, feature selected, and consensus profiles.

The increase in high-throughput cell imaging has resulted in an increase in public imaging data repositories, which has highlighted the need for standardized image-based profiling tools. Repositories such as Image Data Resources^49^ (IDR) and Bioimage Archive^50^ serve as significant sources for raw images. For instance, the IDR repository currently contains 123 studies encompassing 13.7 million images, resulting in 135 terabytes of data.^49^ There is also a growing body of numerical image-based profiling data hosted on other generalist repositories like Zenodo, Figshare, and GitHub. Furthermore, the Cell Painting Gallery on the Registry of Open Data (RODA), hosted by AWS, currently contains over 650 terabytes of both image and image-based profiling data across more than two dozen large-scale image-based profiling datasets.^31^ In fact, many of the datasets in the Cell Painting Gallery contain image-based profiles generated by Pycytominer.^32^ Datasets downloaded from public imaging data repositories can integrate into existing image-analysis workflows, which use Pycytominer to create image-based profiles for subsequent biological discovery and hypothesis testing (Figure 3A).

The image-based profiling pipeline uses and generates several different intermediate data types, which we refer to as “data levels”, as established by the LINCS consortium (Figure 3B).^51,52^ Level 1 data are output by microscopy imaging and early image analysis steps (e.g., illumination-corrected images), and represent the rawest data type. These data are often analyzed directly by deep learning models that process pixels.^53–55^ Level 2 data are extracted profiles of image-based features that are output following single-cell segmentation and/or by deep learning models (e.g., DeepProfiler^33^) and other feature extraction tools (e.g., CellProfiler^43,44^). This is where Pycytominer comes in: Pycytominer generates data levels 3, 4a, 4b, and 5, which represent aggregated, normalized, feature selected, and consensus data, respectively (Figure 3C). Level 4 data are the most common profiles analyzed downstream, but the level 5 data are often used as a consensus phenotypic signature (sometimes referred to as a “fingerprint”) in large-scale drug screens. Scientists can use level 5 data to quickly compare an unknown compound to existing libraries to infer which compound mechanisms may be most similar.^51^ Furthermore, Pycytominer users have the option of skipping the aggregation step to normalize single-cell profiles to perform single-cell analyses. Pycytominer outputs all data levels in standardized formats including comma-separated values (CSV), tab-separated values (TSV), and Parquet files.^56^ These formats are widely recognized and easily used by other software packages for downstream analysis.

### Using Pycytominer with the image-based profiling recipe

**Figure 4.**
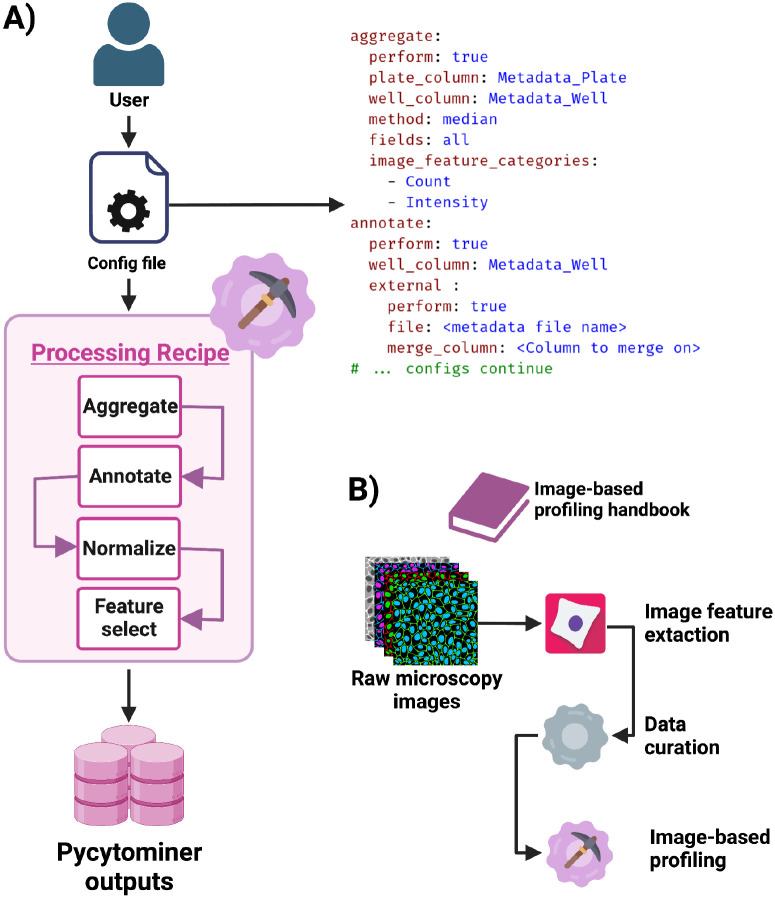
Pycytominer recipe for image-based profiling. **(A)** Users can configure a profiling recipe to customize Pycytominer implementation of image-based profiling steps. Users interact with the profiling recipe through a configuration file provided in yaml format, to parameterize each function within the Pycytominer workflow. **(B)** We have also written an image-based profiling handbook available at https://cytomining.github.io/profiling-handbook/, which documents all steps in a full image analysis workflow, which includes the image-based profiling recipe.

Pycytominer offers a flexible API that enables users to string functions together to perform image-based profiling (Figure 4). The cytomining/profiling-recipe GitHub repository^57^ contains a workflow that incorporates many image-based profiling functionalities provided by Pycytominer (Figure 4A). Users can modify the configuration file, enabling precise control over specific analysis parameters to align with their specific research questions (Figure 4B). This control not only enhances customization but also supports data provenance and reproducibility by providing a simple interface for understanding the exact analytical methodologies used. Moreover, we have detailed all these practical steps in our profiling handbook (Figure 4C).^58^ Starting from raw microscopy images, the image-based profiling handbook outlines a step-by-step process for delivering analysis-ready image-based profiles.

## Discussion

We developed Pycytominer to serve the growing demand for reproducible, consistent, and open-source image-based profiling. The absence of a standardized approach gives rise to a range of issues that each stem from inconsistent analysis and the tendency to duplicate efforts unnecessarily. Pycytominer reduces analytical inconsistencies and promotes uniformity and reliability in analyses across our field. Furthermore, a rigorous testing framework ensures high code quality with minimal risk of introducing errors.

Pycytominer offers an intuitive API that can seamlessly integrate into diverse workflows, ensuring straightforward implementation and customization for various cell types, microscopy methods, assay conditions, and data sources. While this balance between flexibility and modularity promotes a standardized and reliable framework, it also provides a community pathway for future method development and other technical enhancements. Community collaboration is at the center of Pycytominer’s development. Clear contribution guidelines facilitate participation from the open-source Cytomining community, which is focused on developing image-based profiling tools and methods. This collaborative approach ensures ongoing improvements and the validation of Pycytominer’s functionality.

However, Pycytominer is not without its limitations. One notable constraint is that it is written in Python, which may exclude users proficient in other programming languages and necessitates integrating their analytical pipelines into Python. To address this, our future roadmap includes more containerization and the addition of command line interface (CLI) options, which will broaden access and offer multilingual support. Second, there may be more optimal image-based profiling methods not yet discovered. In anticipation of these future developments, we have deliberately designed the Pycytominer API, profiling recipe, and testing framework with modularity in mind. This approach allows for the easy incorporation of new methods as they surpass the current state of the art. Third, it is important to note that Pycytominer focuses on a specific segment of the entire image analysis pipeline. Consequently, users are required to be proficient in other software for preliminary processing steps like quality control and segmentation. This decision to concentrate on core image-based profiling functionality comes with drawbacks, but was made to simplify software maintenance and foster direct image-based profiling innovation.

Looking to the future, Pycytominer is poised to play an important role as an integral tool for image-based profiling. With a steadfast commitment and a growing community consistently contributing new and optimized functionality, Pycytominer offers a reliable and standardized toolkit that empowers researchers to unveil new insights in multiple fields from cell biology to drug discovery.

## Figures and Tables

**Table 1.** Pycytominer’s modules and focus. The table outlines Pycytominer’s core functions and their focused use in image-based profiling.

Module	Focus
**Aggregation**	Provides multiple options for combining single-cell profiles (e.g. by well, by perturbation).
**Normalization**	Provides multiple options for ensuring consistent feature scaling to accommodate diverse feature distributions in both single-cell and aggregated profiles.
**Feature Selection**	Identifies and selects the most informative features, thereby reducing dataset noise and improving the accuracy of biological results.
**Batch Correction**	Identifies and corrects batch effects through methods like sphering. This process mitigates unwanted technical influences in datasets, especially in cell morphology data derived from multiple experimental batches.
**Cyto Utilities**	Provides custom functionality for analyzing and visualizing CellProfiler data, offering features such as the Collate function to centralize data into a single SQLite file, Gene Cluster Text (GCT) file generation for web-based visualization, and the extraction of cell locations within images.

## Data Availability

Pycytominer is an open-source project and its source code can be viewed and downloaded from: https://github.com/cytomining/pycytominer Pyctominer’s installation and usage documentation is available at: https://Pycytominer.readthedocs.io/ The image-based profiling Handbook can be found: https://cytomining.github.io/profiling-handbook/01-overview.html A full pipeline example for Pycytominer is available at: https://github.com/cytomining/pipeline-examples A tutorial on how to conduct single-cell profiling is available at: https://Pycytominer.readthedocs.io/en/latest/walkthroughs/single_cell_usage.html

## Highlights

- Image-based profiling refers to the set of bioinformatics procedures for processing high-dimensional single-cell features extracted from microscopy images.
- Image-based profiling is a rapidly evolving field with new methods, processing steps, and algorithms.
- Pycytominer is a robust and fully tested Python software package with a consistent API.
- Pycytominer contains complete functionality for processing image-based representations that have already been extracted from microscopy images.
- Pycytominer processes classical or deep learning-based features derived from tools like CellProfiler and DeepProfiler.
- We recommend procedures to ensure reproducibility of Pycytominer pipelines and to maximize the value of image-based profiling data.
