# Supplementary material for: Reproducible image-based profiling with Pycytominer
Source: ArXiv. 2024 Jul 2:arXiv:2311.13417v2. Originally published 2023 Nov 22. Preprint. [Version 2] (PMC10690292)
Supplement: Supplement 1 [file NIHPP2311.13417v2-supplement-1.pdf]

Supplementary information

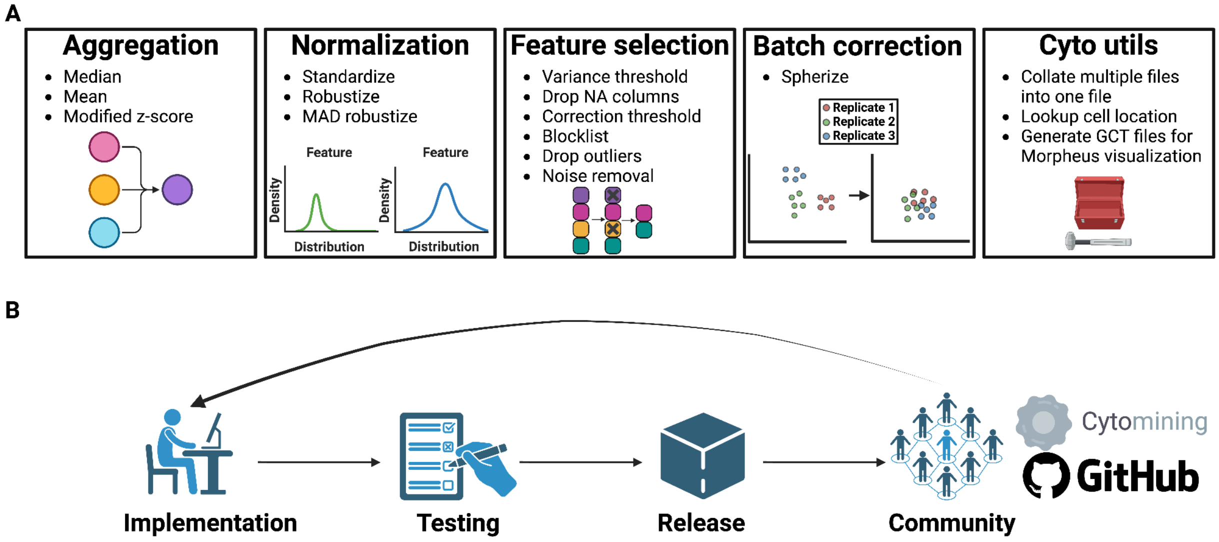

Supplementary Figure 1. *Pycytominer's core Application Programming Interface (API) and software practices.*

(**A)** Pycytominer performs five fundamental functions, each implemented with a simple and intuitive API. Each function enables a user to implement various methods for executing operations. **(B)** The open-source Pycytominer community supports best development practices to facilitate code effectiveness and longevity.

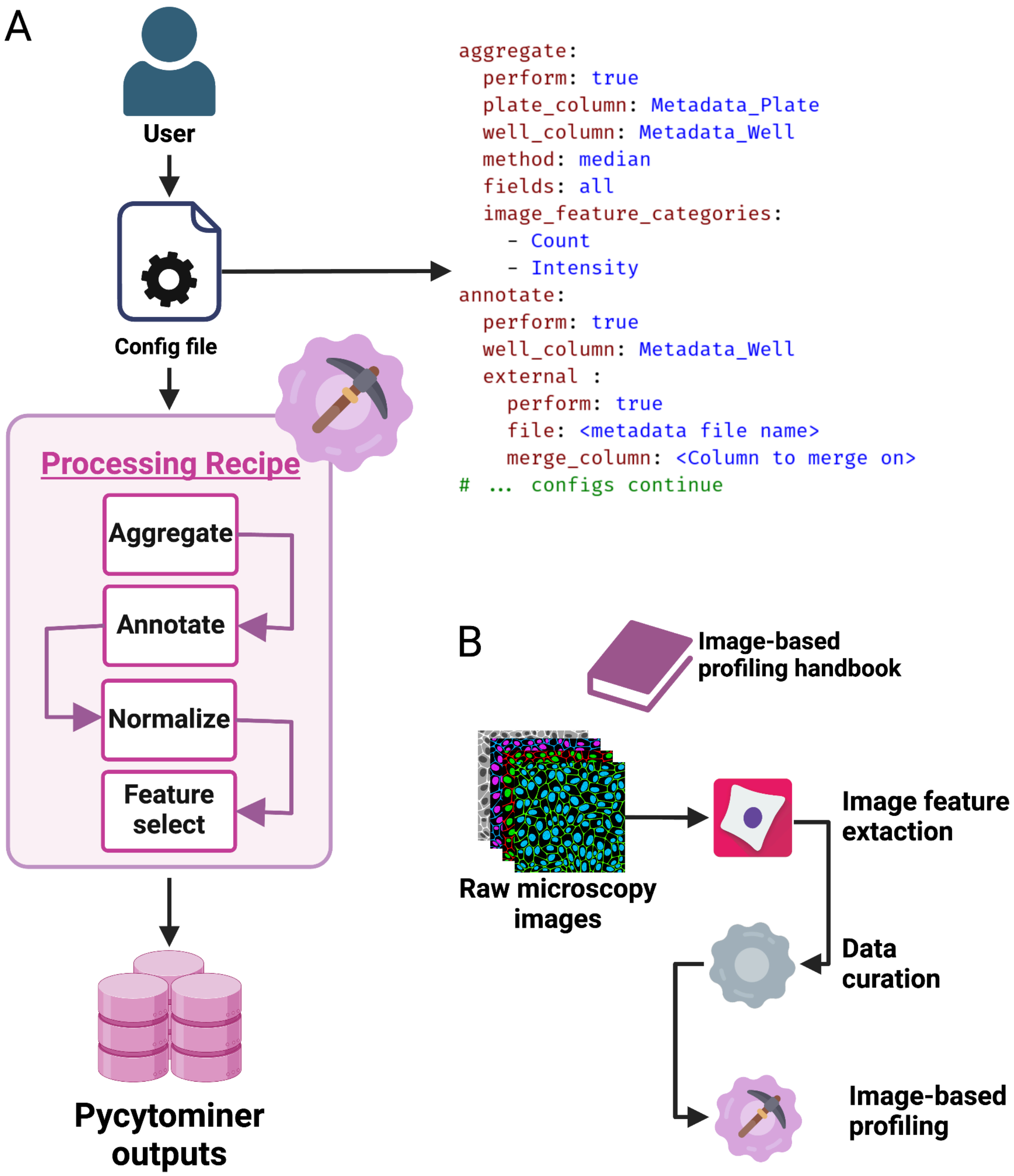

Supplementary Figure 2. *Pycytominer recipe and handbook for image-based profiling.*

(A) Users can configure a profiling recipe to customize Pycytominer implementation of image-based profiling steps. Users interact with the profiling recipe through a configuration file provided in yaml format, to parameterize each function within the Pycytominer workflow. **(B)** We have also written an image-based profiling handbook available at https://cytomining.github.io/profiling-handbook/, which documents all steps in a full image analysis workflow, which

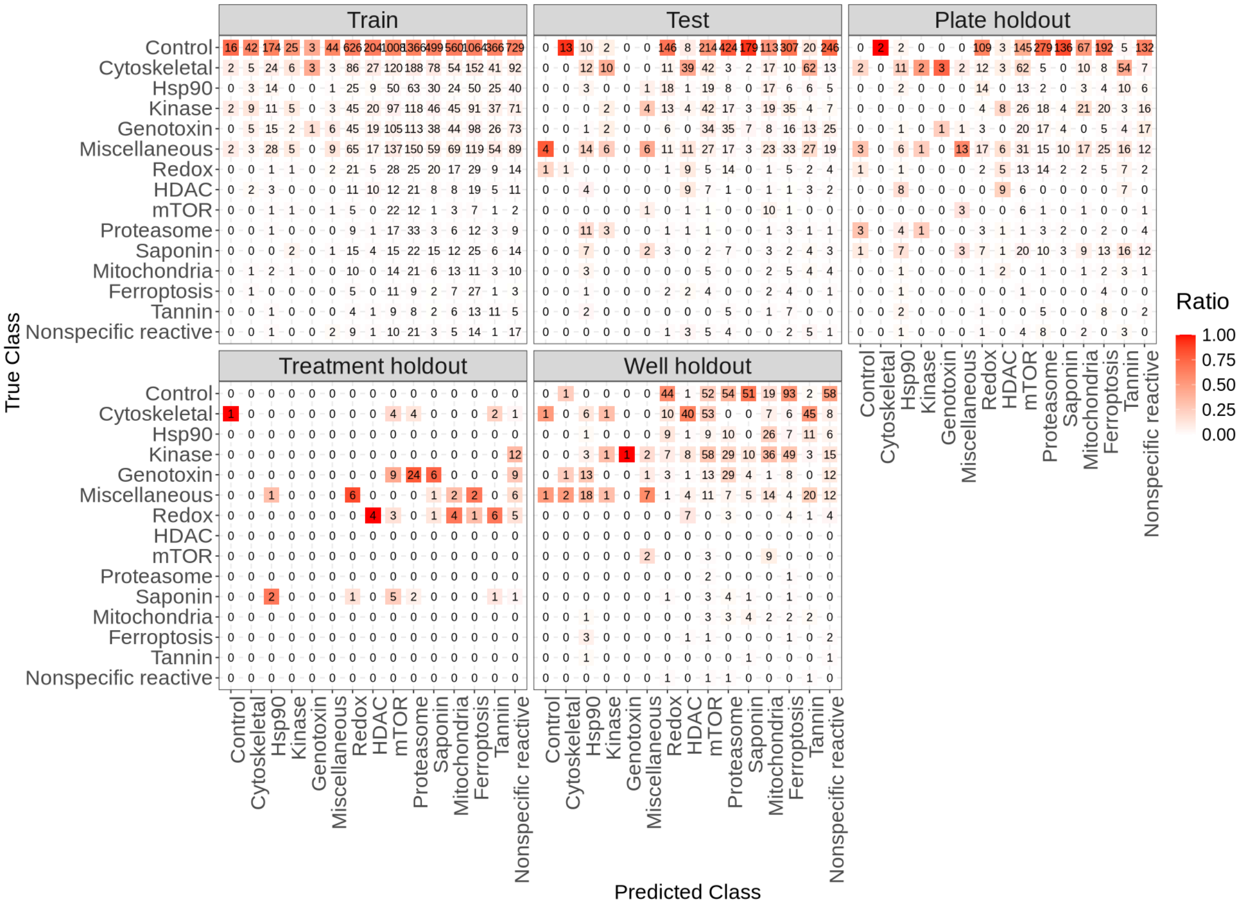

Supplementary Figure 3. *Shuffled multi-class logistic regression model predictive performance.*

The confusion matrix demonstrates the model's ability to predict cell injuries across various data splits and holdouts. The red color gradient represents the ratio of the count over the true class label.

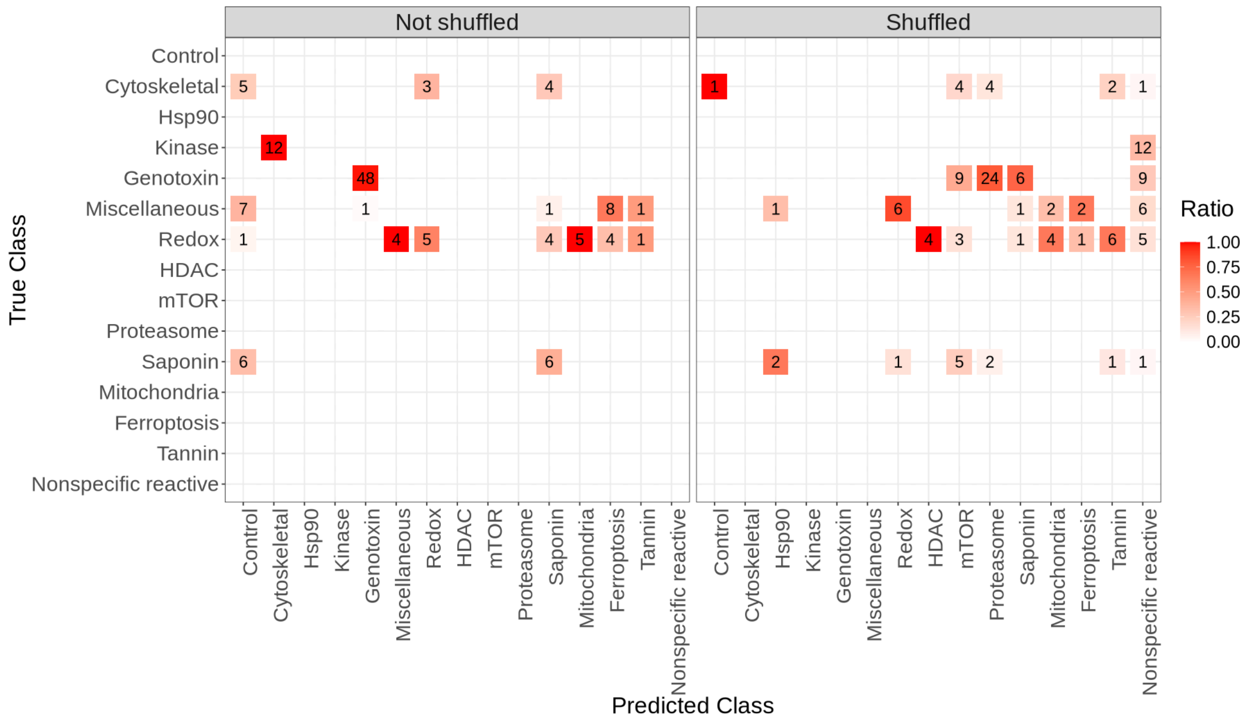

Supplementary Figure 4. *Comparison of multi-class logistic regression model predictive performance for treatment holdouts.*

The confusion matrix evaluates the predictive performance of the Not Shuffled model (left) versus the Shuffled model (right) using only the treatment holdout dataset. The red color gradient corresponds to the ratio, indicating the count over the true class label.

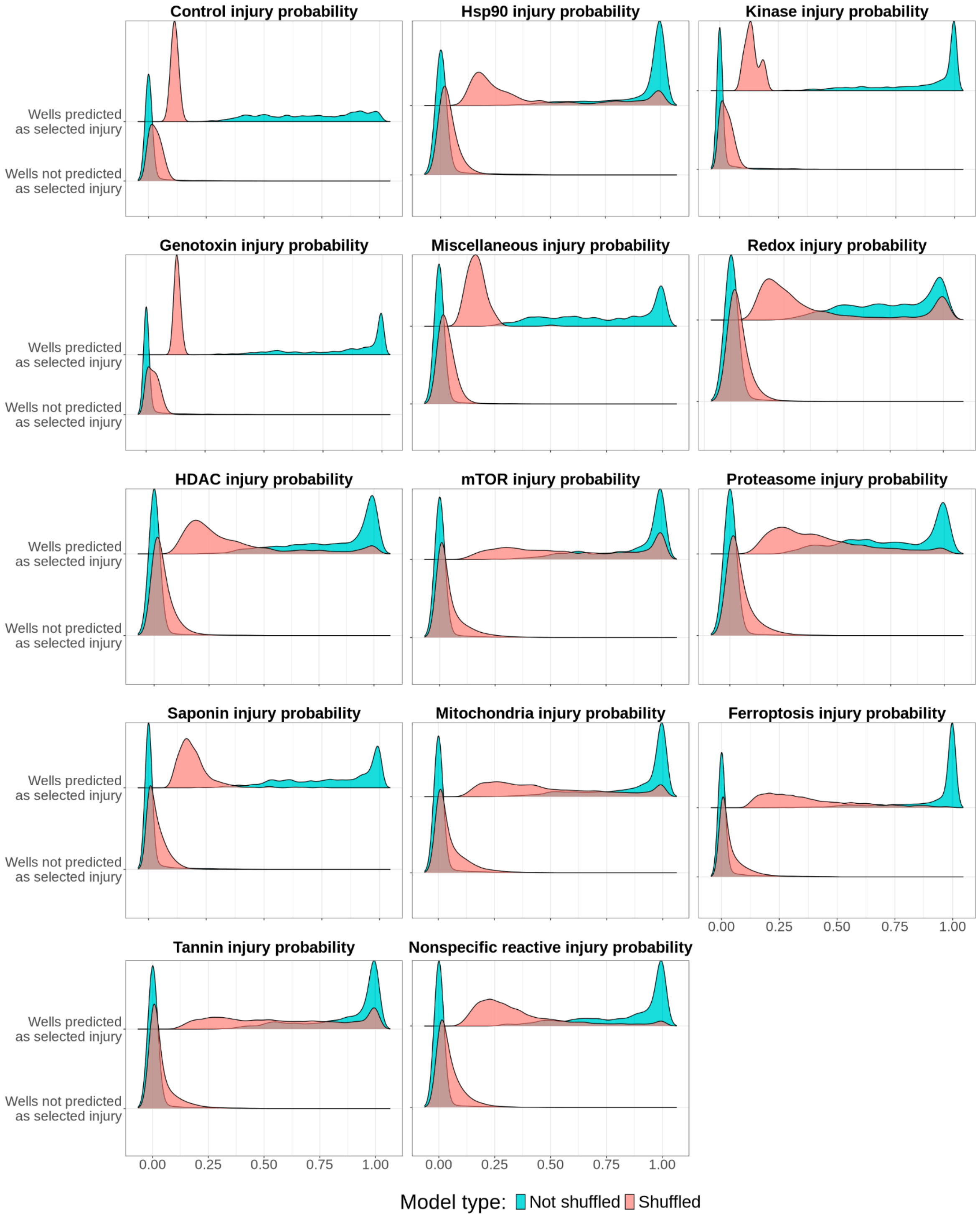

Supplementary Figure 5. *Predicting all other cellular injury types in JUMP data.*

Probability distributions generated by our model for predicting each injury type in the Joint Undertaking in Morphological Profiling (JUMP) Pilot data (CPJUMP1). Per facet, the upper curves indicate wells predicted to have the selected injury (with the top probability), while the lower curves indicate wells not predicted to have the selected injury (anything but top probability). The shuffled model shows lower top probabilities and higher other probabilities compared to the not shuffled model, which shows high model confidence for most injury types, similar to our ground truth performance (see **Figure 2D**).

Software	Language	Key Features	Limitations
Pycytominer	Python	Comprehensive functionality for image-based profiling.<br><br>User-friendly API and documentation.<br><br>Actively maintained and supported.<br><br>Integrates with popular data processing libraries.	Requires proficiency in Python for integration with workflows.<br><br>Pycytominer focuses on image-based profiling, thus necessitating both upstream image analysis and downstream analyses.
BioProfiling.jl	Julia	Implements best practices for image-based profiling.<br><br>Fast performance due to Julia's compilation.	Limited user base and community support compared to Python.
StratoMineR	Web-based	Designed for analyzing high-content screening experiments.<br><br>Graphical user interface for ease of use.	Commercial tool with limited customization options.
Squidpy	Python	Specialized for spatial data analysis.<br><br>Integrates with scanpy for single-cell analysis.	Focuses on cell-type identification and gene expression.<br><br>Limited functionality for general image-based profiling.
PhenoRipper	MATLAB	Rapid feature extraction and data visualization.<br><br>Designed for high-content screening data.	Requires MATLAB; last official update was in 2011.<br><br>Very little options to process image-based profiles.

Supplementary Table 1. *Overview of image-based profiling software tools*

Well-known image-based profiling software tools, highlighting their key features and limitations.

Cellular Injury	Total Wells	Training Wells	Testing Wells	Plate Holdout Wells	Treatment Holdout Wells	Well Holdout Wells
Control	9855	6726	1682	1072	0	375
Cytoskeletal	1472	881	221	181	12	177
Ferroptosis	96	66	16	6	0	8
Genotoxin	944	590	147	73	48	86
HDAC	168	110	28	30	0	0
Hsp90	552	334	84	54	0	80
Kinase	1104	600	150	120	12	222
Miscellaneous	1304	806	201	172	18	107
Mitochondria	144	92	23	12	0	17
Nonspecific reactive	128	84	21	19	0	4
Proteasome	144	94	23	24	0	3
Redox	312	172	43	54	24	19
Saponin	288	131	33	102	12	10
Tannin	96	60	15	18	0	3
mTOR	96	56	14	12	0	14

Supplementary Table 2. *Overview of data splitting process for cell injury dataset*

We categorized the cell injury dataset into distinct subsets for model training, all stemming from the "Total Wells" category (green). "Train Wells" and "Test Wells" were used for model training and testing, respectively (orange). Additionally, we used three holdout sets (pink) to evaluate model performance in never-before-seen plates, treatments, and wells.

Supplementary Table 3. *Cell injury model prediction and probability scores for the JUMP-CP dataset*

This table contains all the predictions made by our model for each well in the CPJUMP1 dataset. Each prediction is accompanied by a probability score indicating its confidence level.
